# Improvements in 4th graders' task behavior after physical activity: mediation by inhibition?

**DOI:** 10.3389/fcogn.2024.1362636

**Published:** 2024-03-06

**Authors:** Christina Hubertina Helena Maria Heemskerk, Claudia M. Roebers

**Affiliations:** Developmental Psychology, Institute of Psychology, University of Bern, Bern, Switzerland

**Keywords:** physical activity, executive functions, inhibition, on-task behavior, off-task behavior, primary school, mediation, acute effects

## Abstract

**Introduction:**

This study aimed to investigate if the acute effects of a physical activity (PA) break on the on-task and off-task classroom behavior of primary school children are mediated by inhibition. Combining arousal theory and the cognitive stimulation hypothesis, we employed a 15-min intermittent PA protocol aiming at high-intensity with cognitive demands. We were interested in the effects of PA in real-life settings and in a feasible and sustainable manner for teachers. Thus, the PA session was short and all data collection carried out in ecologically valid school and classroom environments.

**Methods:**

Fifteen 4th grade classes were randomly assigned to the experimental group (EG; eight classes, *n* = 120) or waitlist control group (WCG; seven classes, *n* = 91). Participants were observed during normal classroom lessons for 25 min before and after the PA break (EG) or a business-as-usual lesson (WCG) and completed the Hearts and Flowers task, a task measuring primarily inhibition, once per observation block. We analyzed the effect of PA on inhibition with ANOVA and the effect on behavior and mediation effect with logistic multilevel models.

**Results:**

The PA break positively affected inhibition with a small effect. Active off-task classroom behavior was higher at post-test in the WCG, but not the EG. Of practical importance, intercept-slope interactions indicated that those with higher levels of off-task behavior at pre-test experienced greater benefits of the PA breaks. No significant mediation of the effect of PA on task-related behaviors via inhibition was found.

**Discussion:**

In conclusion, a time-efficient PA break can improve inhibition and off-task classroom behavior in primary school children. Although these effects occur concurrently, they appear to be independent of each other.

## 1 Introduction

Physical activity (PA) has been linked to improvements in cognitive measures in youth, including task-related behavior during lessons (e.g., Daly-Smith et al., 2018; Masini et al., 2020), academic achievement (e.g., Peiris et al., 2022; Petrigna et al., 2022; Muntaner-Mas et al., 2023), and executive functions (EF) (e.g., Berrios Aguayo et al., 2022; Drozdowska et al., 2022). EF are higher cognitive functions, including the three basic components working memory (holding information in mind and mentally manipulating it), inhibition (ignoring task-irrelevant stimuli and suppressing impulsive behavior), and cognitive flexibility (switching between the rules and requirements relevant to changing tasks) (Miyake et al., 2000; Diamond, 2013). Although improvements in task-related behavior after PA have been suggested to be the result of improved inhibition and impulse control (e.g. Vogan et al., 2018; Mahar, 2019), this has not been extensively researched in studies which investigate the concurrent *acute* effect of PA on task-related behavior and inhibition, as well as the relations between these two outcomes.

Acute PA has been suggested to affect cognitive functions via arousal theory and the cognitive stimulation hypothesis. These pose that PA leads to stimulation of brain areas, which increases the availability of cognitive resources in these areas during subsequent cognitive tasks (Lubans et al., 2022). Additionally, PA leads to an upregulation of neurochemicals important to cognition such as norepinephrine, also known as the monoamine enhancement theory (Meeusen, 2006; Hötting et al., 2016; Veraksa et al., 2021; Lubans et al., 2022). Finally, the attention hypothesis assumes that PA leads to more efficient allocation of cognitive resources and helps to overcome interference, thus improving task-related behavior in the classroom (Mavilidi et al., 2022).

Precise specifications of the optimum dose of PA are still under investigation and recently, two dimensions of acute PA have received particular attention in the literature: intensity [more specifically, vigorous PA (VPA), where heart rate is above 70% of maximum (Wassenaar et al., 2021)] and cognitive engagement, which we will address next.

VPA has been found to elicit cognitive as well as physical health benefits. In a meta-analysis, Moreau and Chou (2019) reported small positive effects of VPA on inhibition (ES = 0.27). Moreover, the authors concluded that short bouts of just 6–10 min of VPA were sufficient to enhance inhibition (ES = 0.24), making VPA particularly time-efficient. Importantly, intermittent short exercise bouts have been suggested as a more effective way to target children's cognition, as these more closely mimic their naturally occurring patterns of activity (Martins et al., 2022). In a meta-analysis of high intensity interval training (HIIT) interventions—by its very nature an intermittent exercise protocol, consisting of short bouts of very high intensity exercise at >85% of maximal heart rate—Leahy et al. (2020) found even greater positive effects [standardized mean difference (SMD) = 0.50] of intermittent VPA for general EF. The addition of cognitive demand during PA is thought to particularly enhance subsequent cognition, as this type of PA more strongly activates the areas of the brain which are involved in higher-order (executive) cognitive functions (Leahy et al., 2020; Becker et al., 2023; Li et al., 2023).

Turning our focus to the effects of PA on ecologically valid measures of on-task classroom behavior, we also find positive results (Masini et al., 2020). Task-related behavior broadly falls into two categories: being on-task or off-task. On-task behavior is mostly defined as displaying task-relevant actions and following the teacher's instructions for completing the task at hand, whereas off-task behavior is any behavior not related to completing the learning task (e.g., Ma et al., 2014; Mavilidi et al., 2020; Godwin et al., 2022; Heemskerk et al., 2022a).

A range of programmes of PA breaks have found improvements in on-task behavior and/or reductions of off-task behaviors, e.g., TAKE10!®(Goh et al., 2016), FUNtervals (Ma et al., 2014, 2015), ACTI-BREAK (Watson et al., 2019). A recent meta-analysis reports a large pooled effect size (standardized mean difference of 1.15) for acute PA, and points toward arousal and attention theory (Mavilidi et al., 2022). Likewise, a recent systematic review by Ruhland and Lange (2021) also reports overall positive effects of PA breaks on on-task behavior. Critically, the content of the PA bout was found to be important in a studies by Heemskerk et al. (2020) and Mavilidi et al. (2020)—both concluding that high levels of physical activity with cognitive demands was most conducive to improving on-task behavior.

Being on-task during lessons requires EF; in particular inhibition is necessary to filter out irrelevant stimuli and maintain focus on one's task whilst ignoring the many task-unrelated behaviors possible in a room full of peers. Yet, to date, little is known about the exact contribution of EF components—including inhibition—to task-related behavior. The majority of recent research has been conducted in pre-K and kindergarten (e.g., Brock et al., 2017; McCoy et al., 2022; Nesbitt and Farran, 2022). In an older sample of second-graders, Heemskerk and Roebers (2023) report that inhibition and switching both related to on-task behavior (β = 0.19 and β = 0.17, respectively). This suggests that EF and task-related behavior do share some observable and unobservable cognitive demands and processes. However, evidence of the link between EF and task-related behavior is scarce for school-aged children, especially beyond the first primary school years.

And, although inhibition and task-related behavior are both affected by PA, specific knowledge of whether the improvements in behavior occur via improvement in inhibition remains lacking, which is where this study aims to contribute to the literature. The present study goes beyond previous studies of the effects of activity breaks on cognition by investigating the effects of a PA break on both EF and task-related behaviors, as well as the interrelations between EF and task-related behavior. Combining arousal theory and the cognitive stimulation hypothesis, the optimum point of arousal could be reached in a short time using VPA. Adding cognitive demands to the exercise would stimulate the appropriate areas of the brain for enhanced cognitive function and on-task behavior. Thus, the present study employed a short, intermittent PA protocol aiming for high-intensity with cognitive demands to investigate the effects of this PA break on inhibition and task-related behavior, as well as a possible mediation of the effects on task-related behavior by inhibition.

We posed the following research questions:

- Does participation in a single bout of cognitively demanding PA aimed at high-intensity lead to better inhibition?
- Does participation in a single bout of cognitively demanding PA aimed at high-intensity improve subsequent task-related behavior?
- Is inhibition related to task-related behavior?
- Does EF performance mediate the acute effect of cognitively demanding PA aimed at high-intensity on task-related behavior?

Based on monoamine enhancement theory (Unsworth and Robinson, 2017; Veraksa et al., 2021), arousal theory and the cognitive stimulation hypothesis (Leahy et al., 2020; Lubans et al., 2022), and the attention hypothesis (Kao et al., 2017; Ludyga et al., 2017; Mavilidi et al., 2022), we expected our PA protocol to have a positive acute effect on inhibition and on task-related behavior. Moreover, based on the current literature, we expected small correlations between on-task behavior and inhibition (Nesbitt et al., 2015; Nesbitt and Farran, 2022; Heemskerk and Roebers, 2023). As no previous reports of the mediation of PA effects on task-related behavior via EF were found, those analyses were carried out in an exploratory way, without hypotheses.

## 2 Materials and methods

### 2.1 Sample and recruitment

**Figure 1:**
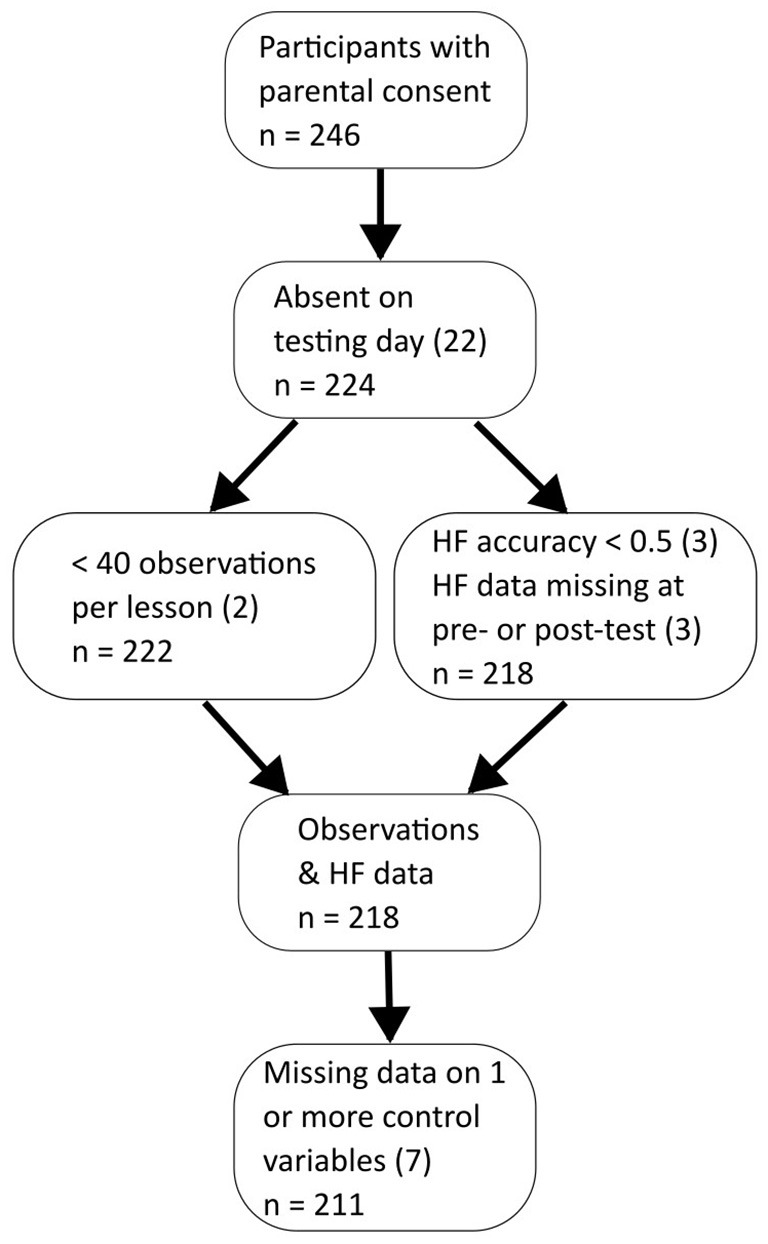
Overview of participant exclusions.

Ethical clearance was provided by the University of Bern. Data were collected between October and December 2022 in 15 fourth grade classrooms. Teachers were invited to participate in the study with their classes, and children with informed parental consent were allowed to take part (*n* = 246). A total of 211 children had valid data on all variables and were included in analyses. A flowchart of data exclusions is provided in Figure 1. Classrooms were randomly assigned to one of two groups; the experimental group (EG), taking part in the 15-min PA session (*n*_classroom_ = 8, *n*_participants_ = 120, 51.7% female, *M*_age_ = 126.2 m, SD_age_ = 7.4), or the waitlist control group (WCG), receiving their “business-as-usual” classroom lesson (*n*_classroom_ = 7, *n*_participants_ = 91, 54.9% female, *M*_age_ = 128.1 m, SD_age_ = 8.0; teachers of WCG classes received a video of the intervention session to use with their class after data collection was completed). The intervention and control group did not differ in age (*W* = 6,082, *p* = 0.146), sex ($\chi_{\left(1\right)}^{2}$ = 0.111, *p* = 0.739), highest level of parental education (*W* = 2,628, *p* = 0.205), BMI (*W* = 5,443, *p* = 0.970), fluid intelligence [odd-one-out task from the RIAS (Reynolds and Kamphaus, 2003; Hagmann-von Arx and Grob, 2014), *W* = 5377, *p* = 0.851], or working memory [reverse color span (Zoelch et al., 2005), *t*_(191.29)_ = 0.73, *p* = 0.465]. A full overview of the sample characteristics is available in Table 1.

**Table 1:** Sample descriptives, by condition.

**Variable**	**WCG**	**EG**	**Group comparison**	***p*-value**
n	91	121		
Male—*n* (%)	42 (46.2)	58 (47.9)	$\chi_{\left(1\right)}^{2}$ = 0.111	0.739
Age in months	128.2 (8.0)	125.9 (6.9)	*W* = 6,082	0.146
BMI *z*-score	0.36 (1.6)	0.50 (1.7)	*W* = 5,443	0.970
Fluid intelligence	51.3 (12.0)	50.9 (12.4)	*W* = 5,377	0.857
Reverse color span	10.6 (3.1)	10.2 (3.1)	*t*_(191.29)_ = 0.73	0.465
Parental education—*n* (%)	1 (1.1)	3 (2.5)	*W* = 2,628	0.205
No formal schooling				
Obligatory schooling	1 (1.1)	4 (3.3)		
Vocational qualification	27 (29.7)	17 (14.3)		
High school	3 (3.3)	4 (3.3)		
Higher vocational	16 (17.6)	13 (10.8)		
University degree	26 (28.5)	39 (32.5)		
Missing	17 (18.7)	40 (33.3)		

Data are presented as Mean (SD), unless otherwise indicated. WCG, waitlist control group; EG, experimental group.

### 2.2 Procedure

Each class received two visits, one week apart from each other. During the first visit, height and weight measurements were completed, and three cognitive tasks were carried out: the Hearts and Flowers task (HF) as a measure of inhibition (Davidson et al., 2006; Diamond et al., 2007), a backwards color span working memory task (Zoelch et al., 2005), and the Odd-Item-Out task from the RIAS as a measure of fluid intelligence (Reynolds and Kamphaus, 2003; Hagmann-von Arx and Grob, 2014). All tasks were completed on touch screen tablets (Samsung Galaxy Tab A7 and S4, screen size 10.5′′ and 10.4′′, respectively), with instructions and feedback provided via headphones. The children completed the tasks as a whole class, in their usual classroom seats. As they had a tablet each to use, and instructions were programmed into the task, they were able to work at their own pace. The working memory and fluid intelligence tasks were administered as measures of individual differences, whereas the HF was conducted as a familiarization to reduce learning effects from pre- to post-testing at the second visit. At the end of the first visit, socio-economic status questionnaires for parents were handed out and collected at the second visit.

**Figure 2:**
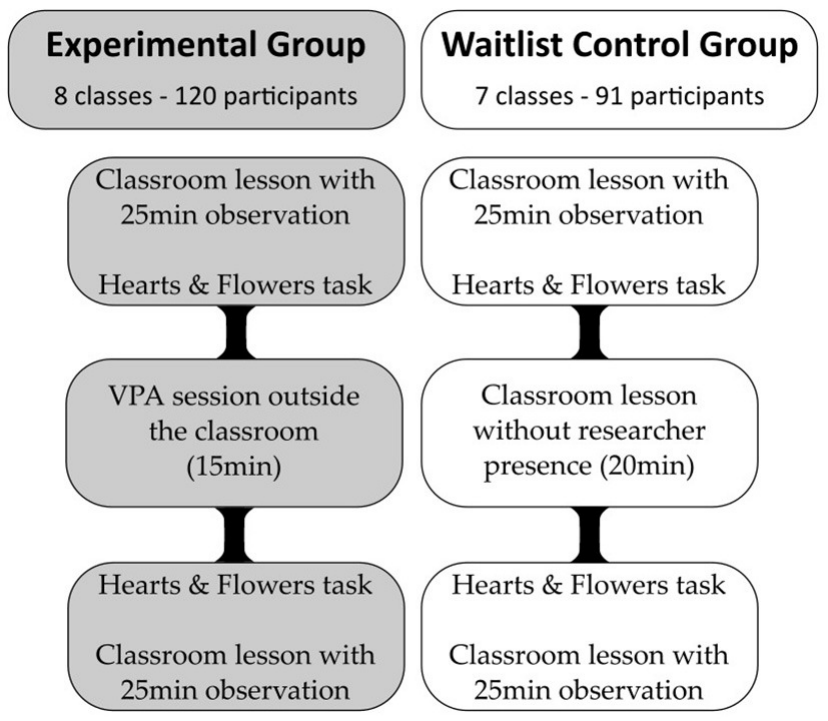
Overview of the data collection procedure.

During the second visit, the research team observed the participants for 25 min during their normal classroom lesson, using the SchoolBehavior app (Heemskerk and Roebers, 2023), available from the Google Playstore and Apple store free of charge. At the end of the lesson, the children completed the HF. Then, EG classes took part in a brief PA session outside the classroom (*M* duration = 14 min53, SD = 1min12), aimed at intermittent high intensity and complexity. Upon return to the classroom, they completed ratings of enjoyment and importance for the sports session in a Qualtrics questionnaire on their tablet. Transitions between the classroom and the PA session took <5 min. Instead of the PA session, the WCG classes continued their classroom lesson, without the research team being present. We did not manipulate the content of the WCG classes during this time as we wanted to investigate the effect of the PA session compared to “business as usual” classroom lessons. Both groups completed the HF once more and were then observed during their classroom lesson for another 25 min. An overview of the protocol for the second visit is presented in Figure 2.

#### 2.2.1 Physical activity session

The PA session was delivered by the first author, who was not present during classroom observations and the completion of the HF. The research team carrying out observations were not present during the PA session. The session took place in a separate area of the school, away from the classroom. Weather permitting, the session was completed in the playground. To make the data collection as ecologically valid as possible, no devices were put on for registering PA intensity. The session content was designed to yield a high heart rate and the researcher delivering the PA session closely monitored the children's participation and correct execution of the exercises, and encouraged all participants to achieve as much VPA as possible.

The session started with a 2-min warm-up of jogging and sprinting on the spot. On cue, side-stepping movements, touching the ground, and jumps were mixed in. Then, the participants were set a 1-min challenge to perform as many bunny hops (touch your hands to the ground and jump as high as you can) whereby each bunny hop was scored with one point. If they synchronized their jump with another participant and did a high ten in the air, they could score ten points. They could only high-ten each of their classmates once, and thus had to move around to score as many points as possible. Participants were told they needed to remember their number of points, as they would still need the number later.

The main part of the session was divided into two sections, focusing first on arm movements, and then on legs. For the arms, four different combinations of movements based on boxercise were used. Each sequence was demonstrated and then repeated multiple times (between three and five repetitions) at high speed, interspersed with three criss-cross jumps. For the legs, four different combinations of criss-cross jumps were included. These were also demonstrated and repeated at high speed, interspersed with a short boxercise arm movement sequence based on the movements learned in the previous part of the session. Again, the number of repetitions varied with each sequence performed. Finally, the participants repeated their 1-min bunny hop challenge, aiming to beat their number of points from the start of the session. As soon as the session was completed, the participants returned to their classroom.

All movement sequences were carried out at high speed, and the frequent jumping ensured that the sequences could achieve as much high intensity PA as possible. During the demonstration of the next sequence, participants had the opportunity to briefly recover, making the PA bursts intermittent. Following suggestions by Tomporowski et al. (2015) and Basso and Suzuki (2017), cognitive challenge was induced by including (1) complex sequences of arm and leg movements (multi-limb sequencing), (2) multi-directional movements whereby they continuously needed to respond to their environment (warm-up), (3) actions requiring control of movement in order to maintain balance and keep up with the pace of the lesson, and (4) changing the combination of the movements with each sequence, so that participants needed to inhibit previously learned sequences, requiring continual focus and preventing them from automating their movements.

### 2.3 Materials and measures

#### 2.3.1 Pre- and post-test

##### 2.3.1.1 Hearts and Flowers

**Figure 3:**
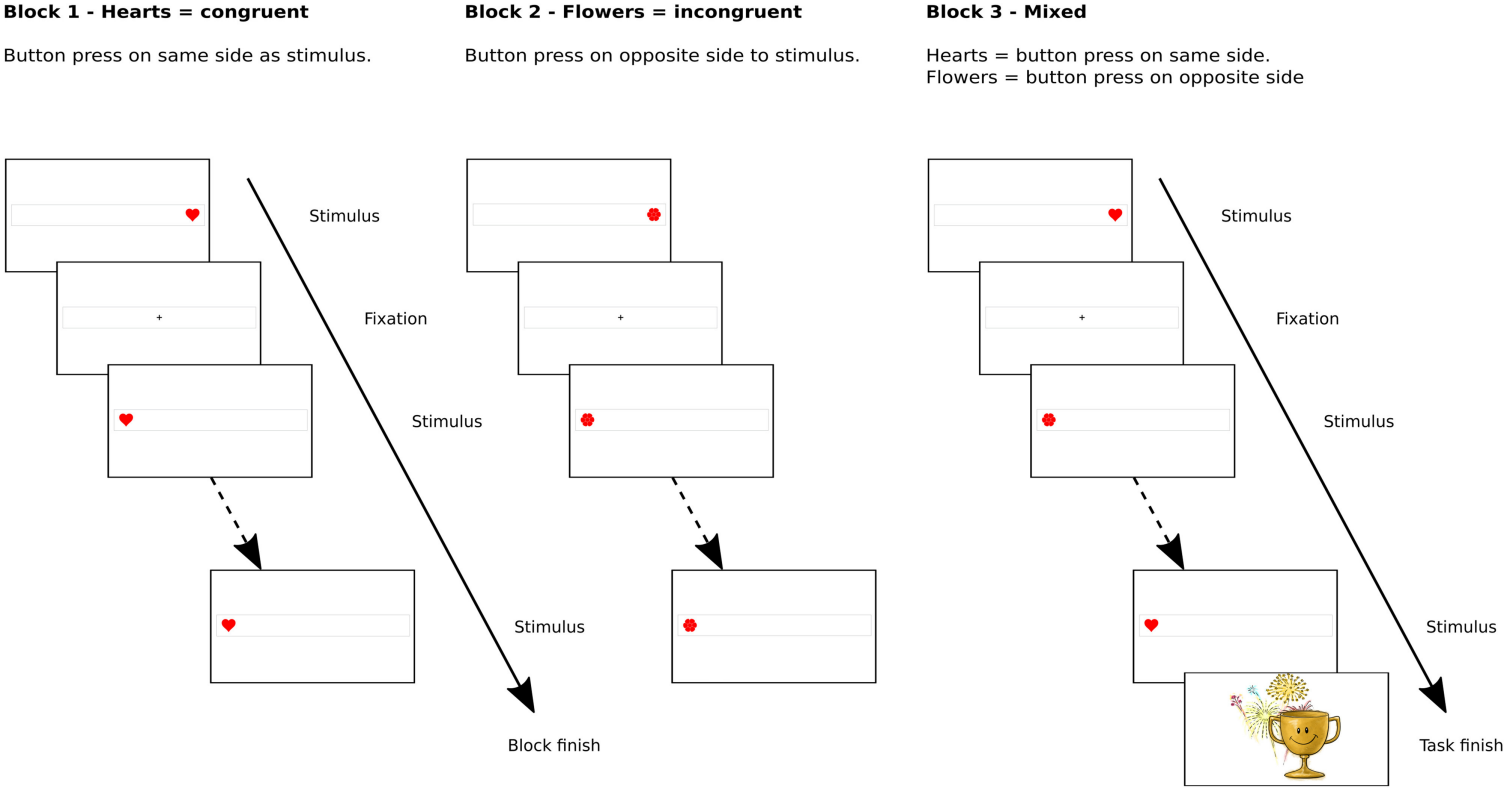
Overview of the HF task.

The Hearts and Flowers task we employed consisted of three blocks (12 congruent trials, 24 incongruent trials, and 36 mixed trials), each preceded by four practice trials, which needed to be solved with 100% accuracy to proceed to the main task. If participants did not succeed in the practice trials, the task halted and additional instructions were provided by a research team member. Children responded to the stimuli by tapping buttons on the tablet's touch screen. In the congruent block, participants needed to tap the button on the same side as where the stimulus (a red heart) appeared. In the incongruent block, the opposite button to where the stimulus (a red flower) appeared needed to be tapped. In the mixed block, if a heart was presented, the button on the same side needed to be tapped, and if a flower was presented (20% of trials), the button on the opposite side needed to be tapped (see Figure 3). The instructions given to participants (via the headphones) can be found in Table 2. Accuracy and response time (RT) were recorded for each trial. Every trial was preceded by a screen with a central fixation cross for 500 ms followed by a blank screen for 500 ms (total ISI 1,000 ms). Stimulus presentation time was 600 ms. Mean time taken to complete the HF was 6.9 min (min = 5.6, max = 10.7).

**Table 2:** Script of hearts and flowers instructions.

**Block**	**Instructions provided**
Hearts—practice	Today we will play the hearts game. When you see a heart on the screen, you must press the button on the same side as the heart. So, if the heart is on the right, like it is now, you must press the right button. You can try this right now. And, if the heart is on the left side, like it is now, you must press the left button. You can try this now Let's practice this at the speed that the game will be. Remember, always press the button on the same side as the heart. This is just a practice, you do not need to rush
Hearts	Well done. Now we'll play the proper the game. Try to press as quickly as you can, but not so fast that you make a mistake. Remember, always press the button on the same side as the heart
Flowers—practice	Now we will play the flowers game. When you see a flower on the screen, you must press the button on the opposite side. So, if the flower is on the left, like it is now, you must press the button on the right. You can try this now. And, when the flower is on the right, like it is now, you must press the button on the left. You can try this now Let's practice this. Remember, always press the button on the opposite side to the flower. This is just a practice, you do not need to rush
Flowers	Well done. Now we'll play the proper game. Try to press as quickly as you can, but not so fast that you make a mistake. Remember, always press the button on the opposite side to the flower
Mixed—practice	We will now play the hearts game and the flowers game together. If you see a heart, you must press the button on the same side as the heart. If you see a flower, you must press the button on the opposite side to the flower Let's practice this. Remember, for hearts, press on the same side, and, for flowers, press on the opposite side. This is just a practice round, you do not need to rush
Mixed	Well done. Now we'll play the proper game. Try to press as quickly as you can, but not so fast that you make a mistake. Remember, for hearts, press on the same side, and, for flowers, press on the opposite side

##### 2.3.1.2 Classroom behavior observations

The SchoolBehavior app (Heemskerk and Roebers, 2023, freely available from app stores for Android and iOS) was used to record observations. A momentary assessment protocol was used with 30-s intervals, meaning that a snapshot assessment of behavior was made every 30 s, and behavior occurring between two observation points was not considered. At each observation point, the participant's behavior was categorized as “on-task,” “passive off-task,” “active off-task,” or “other.” On-task behavior includes all goal-directed behaviors aimed at completing the task set by the teacher. Passive off-task behavior is yawning, stretching, staring, or sleeping in the absence of goal-directed behavior. Active off-task is non-task-related talking or movement. If behavior could not be categorized, or if the participant was out of view from the researcher at the observation point, “other” was recorded. At each observation point, the type of task set by the teacher was also recorded, as this has been shown to influence task-related behavior (Godwin et al., 2013; Heemskerk and Malmberg, 2020; McCoy et al., 2022). Categories for tasks type were “teacher-led,” “independent,” “pair work,” “group work,” and “other.”

Each member of the research team observed up to six participants. The research team was trained in the use of the app and the observation protocol before the start of data collection. Light's Kappa for the research team (six members) was good (0.71). Video materials for training and the full observation protocol are available from the author.

#### 2.3.2 Individual differences

##### 2.3.2.1 Fluid intelligence

We used the German version of the Odd-Item-Out task from the RIAS (Reynolds and Kamphaus, 2003; Hagmann-von Arx and Grob, 2014) as an indication of fluid intelligence. In each presented matrix, participants had to identify the “odd item out” from a set of related stimuli. Four practice matrices were completed at the start, with feedback provided. In the main task, when three consecutive matrices were solved incorrectly, the task automatically ended. For correct answers within 30 seconds participants received two points, for correct answers within 50 seconds they received one point. No points were scored in the practice trials. Possible scores were 0 to 102 points.

##### 2.3.2.2 Working memory

As a measure of working memory, participants completed a backwards color span task based on the task by Zoelch et al. (2005). We created a cover story of a dwarf who lost colored disks from his bag. The children needed to recall the order in which the disks had fallen out of the bag, in reverse order. The first four trials were practice trials, which were repeated if the participant made a mistake. The main task consisted of blocks of six trials per sequence length and started with sequences of two disks. Children progressed to the next block if they answered at least three of the six trials correctly, with the longest possible sequence being seven disks. We calculated the total number of correct trials across the task (excluding practice trials) as our measure of working memory.

##### 2.3.2.3 Anthropometrics

Height was measured to one tenth of a cm with a wall-fixed measuring stick. Children removed their shoes and stood with their back to the measuring stick. Each participant was measured twice, and if the recordings varied by more than half a cm, measurements were repeated until two measurements within half a cm were recorded. Weight was measured to one tenth of a kg using electronic scales (Tanita Innerscan Dual). Participants were weighed twice, and if their recordings differed by more than two tenths of a kg, measurements were repeated until two measurements were within two tenths of a kg were recorded. For both height and weight, the mean of the two measurements was used as the final metric.

### 2.4 Analytic procedures

The analyses were pre-registered on OSF: https://doi.org/10.17605/OSF.IO/ETHXS.

#### 2.4.1 Variable creation

##### 2.4.1.1 Main variables

From the HF data, trials with RT <200 ms or >2,000 ms were removed (3,359 trials, 6.1%). Next, the proportion of correct trials and the mean RT on correct and incorrect trials (separately) were calculated per block, along with the Δ RT for the flowers block. The Δ RT is calculated by subtracting the RT on correct trials in the congruent (Hearts) block from the incongruent (Flowers) block. This is commonly used as a measure of inhibition, as it reflects the additional time required to respond correctly when the incorrect, prepotent (congruent) response needs to be inhibited in favor of the correct (incongruent) response (Camerota et al., 2020). RT for incorrect trials was included in the analyses as a shorter RT on error trials indicates more impulsive responding patterns; responding before the stimulus has been fully evaluated to elicit the correct response (Ambrosi et al., 2019; Roebers, 2022). Participants with an accuracy below chance (<0.5) on any block were removed from the data set (*n* = 3). A further three participants were removed as they did not have data for both pre- and post-test.

A total of 9,146 behavior observations were recorded at pre-test, and 8,963 at post-test. Observations where both behavior and task were coded “other” were removed from the data set (1.75% at pre-test, 1.17% at post-test). For each individual, only lessons where they had at least 40 valid observations were included in the analyses (two participants were removed). A total of 222 participants had valid observation data at pre- and post-test, of whom 218 also had valid HF data. The proportion of observations rated on-task, passive off-task, and active off-task were calculated for use in correlation analyses. We created binary variables for each behavior type (on-task, passive off-task, and active off-task) for use in logistic multilevel models (MLM, see below). Four participants had off-task behaviors of more than 50% at pre- or post-test. We repeated analyses with these participants removed, and found no substantive differences in the results. Thus, we report the results with these participants included to reflect the heterogeneous nature of a naturalistic classroom sample.

##### 2.4.1.2 Control variables

Task recordings (as dummy variable per type of task) were used as control variables in the logistic MLM. Additionally, we calculated the proportions of time working in each task mode and the most frequent task type for each lesson for inclusion in correlations analyses, and as control variables in the mediation models. Height and weight were assessed with the “zscorer” package in R to create a body mass index (BMI) *z*-score for age and sex. This package uses the 2007 reference data from the World Health Organization. BMI *z*-score was used alongside fluid intelligence and working memory as control variables in the analytical models. For the analyses of behavior, sex was included in the control variables. Seven participants had missing data on at least one control variable and were excluded from the analyses, reducing the total sample for analyses to *n* = 211 participants.

#### 2.4.2 Analyses

All analyses were carried out in RStudio (version 4.2.0) and significance was set at 0.05. We analyzed group differences with *t*-tests and Wilcoxon tests, and found no differences in the control variables at pre-test. As accuracy in the HF task was well above 80% in all blocks—the point from which RT is suggested to be a more informative metric of EF than accuracy (Zelazo et al., 2013; Camerota et al., 2019)—we include accuracy in descriptive analyses only. To investigate the relationship between inhibition and task behavior, we report Spearman correlations to allow for the non-normal distribution in the behavior and HF variables. Correlation analyses were performed on the pre-test data only, to avoid interference of intervention effects.

To assess the effect of PA on inhibition, we carried out mixed ANOVAs with Δ RT in the Flowers block as the outcome, condition (EG or WCG) as the between factor and measurement point (pre or post) as the within factor. All ANOVAs were controlled for fluid intelligence, working memory, and BMI *z*-score. The inclusion of sex did not affect the outcome of the analyses, and we report the results of the most parsimonious analyses. We used the package “nlme” (version 3.1–157, RRID:SCR_015655) to allow for non-normal distribution in the outcome variable. As follow-up for significant ANOVA results, pair-wise contrasts with Tukey correction for multiple comparisons in the package “emmeans” (version 1.8.7, RRID:SCR_018734) were used to identify the nature of the significant differences. Effect sizes of ANOVA effects are reported as ω^2^, generated with the “effectsize” package. Effects of ω^2^ ≥ 0.01 are considered small, ω^2^ ≥ 0.06 is considered a moderate effect, and ω^2^ ≥ 0.14 a large effect (Field et al., 2012).

To analyse the effect of PA on behavior, we specified three-level logistic multilevel models (MLM; observation points nested in students, students nested in classes) using the glmer function from the “lme4” package (RRID:SCR_015654). For this approach, we calculated separate models for each behavior type (on-task, passive off-task, and active off-task) using the binary variable for each behavior type as the outcome variable, with condition (EG or WCG), time point (pre or post) and the interaction of condition and time point as the predictors. We allowed for random intercepts and slopes at each level, and included dummy coded task type as a control variable, along with BMI *z*-score, sex. The inclusion of fluid intelligence and backwards color span as control variables did not affect the results, so the more parsimonious models without these control variables are reported. The effect of the intervention on task-related behavior is reported as logit estimates which were subsequently converted to Cohen's *d*, which is considered negligible below 0.2, small between 0.2 and 0.5, moderate between 0.5 and 0.8, and large above 0.8 (Haddock et al., 1998).

**Figure 4:**
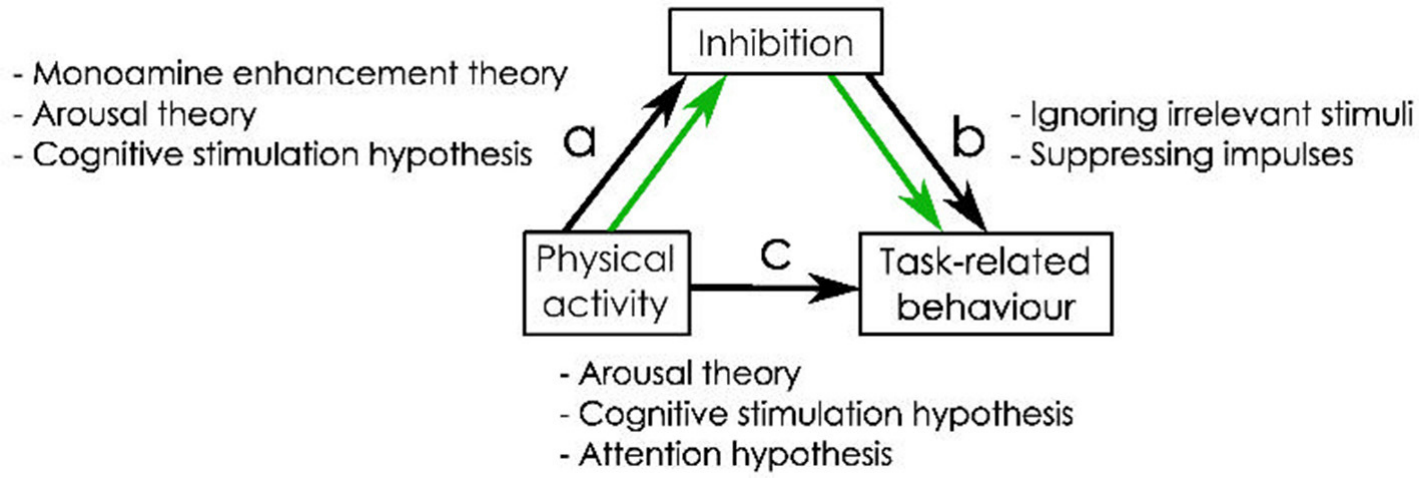
Schematic representation of the direct, indirect, and total effects in mediation.

To assess mediation in the relationship between PA and task behavior, we ran an additional MLM for each behavior type. We added the mediator (Δ RT, scaled) as a predictor to the MLM of task-related behavior. We extracted the total effect of PA on task-related behavior (original model) and the direct effect (model with mediator included). Following MacKinnon et al. (2004), we subtracted the direct effect from the total effect to find the indirect effect (as illustrated in Figure 4). Next, the extent of mediation was assessed with the effect ratio (*P*_*M*_ = indirect effect/total effect) (Shrout and Bolger, 2002). A value of 1 indicates full mediation and a value of 0 no mediation at all. As our variables were not normally distributed, the traditional Sobel method of calculating *p*-values for indirect paths is considered less informative for judging the meaningfulness of indirect and/or mediation effects (Shrout and Bolger, 2002; MacKinnon et al., 2004; Preacher and Hayes, 2008).

A priori sample size calculations in G*Power3 (Faul et al., 2007) indicated that to detect a small effect size with a power of 0.8, a total sample of 200 was required for the ANOVAs of inhibition. For the multilevel analyses of task-related behavior, after accounting for clustering at the participant level by applying a design factor of 2.7 (based on the equation from De Jong et al., 2010), 184 participants were needed. Thus, our sample of 211 participants satisfied these requirements.

## 3 Results

### 3.1 Descriptive statistics

At pre-test, on-task behavior was high in both groups, at 79%. Off-task behaviors occurred in around 10% of observations in both groups. At post-test, on-task behavior occurred in 77 and 78% of observations in WCG and EG, respectively. Passive off-task behavior was reduced to 8% of observations in both groups, whereas active off-task behavior was recorded in 15% (WCG) and 14% (EG) of observations. HF data show similar trends at pre- and post-test with high accuracy scores throughout (above 90%), but consistently the highest accuracy in the Hearts block (congruent) and lowest accuracy in the Mixed block. RT was shortest in the Hearts block and longest in the Mixed block. Table 3 gives an overview of the descriptives of the outcome variables at pre-test. We found a significant difference in the Δ RT in the Flowers block at pre-test, but a follow-up ANOVA indicated that this difference between the two groups did not significantly affect the Δ RT scores at post-test (*p* = 0.847).

**Table 3:** Executive function and behavior descriptives at pre-test, by condition.

**Variable**	**WCG**	**EG**	**Group comparison**	***p***
	Task-related classroom behavior^*a*^			
On-task	0.79 (0.13)	0.79 (0.15)	*W* = 5,785	0.879
Passive off-task	0.11 (0.08)	0.09 (0.07)	*W* = 6,325.5	0.309
Active off-task	0.11 (0.10)	0.12 (0.13)	*W* = 5,636	0.634
	Hearts and Flowers			
Hearts accuracy	0.98 (0.05)	0.99 (0.02)	*W* = 5,933	0.806
Hearts RT correct trials in ms	653.3 (111.6)	631.5 (103.9)	*W* = 6,351.5	0.284
Hearts RT incorrect trials in ms	568.4 (203.1)	451.9 (201.0)	*W* = 255	**0.032**
Flowers accuracy	0.96 (0.05)	0.96 (0.05)	*W* = 5,536.5	0.467
Flowers RT correct trials in ms	739.5 (128.7)	746.2 (110.9)	*W* = 5,207	0.161
Flowers RT incorrect trials in ms	643.4 (181.9)	666.9 (192.9)	*W* = 1,909	0.479
Flowers Δ RT in ms	84.6 (72.5)	119.5 (82.5)	*t*_(204.42)_ = −3.26	**0.001**

Data are presented as Mean (SD). WCG, waitlist control group; EG, experimental group. ^*a*^Task-related classroom behavior is reported as the proportion of the lesson spent displaying the behavior. Significant *p*-values in bold.

On-task behavior strongly and negatively correlated with both passive and active off-task behavior, but the two types of off-task behavior did not correlate with each other. At pre-test, higher proportions of independent work in a lesson were related to lower proportions of on-task behavior (ρ = −0.15, *p* = 0.024) and more recordings of active off-task behavior (ρ = 0.17, *p* = 0.015). On the other hand, more group work related to fewer occurrences of active off-task behavior (ρ = −0.17, *p* = 0.011). The full correlation matrix of pre-test behavior and task type is presented in Table 4.

**Table 4:** Correlation matrix of observation variables and inhibition measures at pre-test.

		**On-**	**Passive**	**Active**
Behavior	On-task	1		
	Passive off-task	−0.64^***^	1	
	Active off-task	−0.76^***^	0.09	1
Task proportion	Independent	−0.15^*^	−0.02	0.17^*^
	Pairs	−0.06	−0.03	0.13
	Groups	0.11	0.04	−0.17^*^
	Teacher-led	0.11	0.01	−0.09
Dominant task type	Independent	−0.06	−0.05	0.08
	Pairs	0.04	−0.02	−0.01
	Teacher-led	−0.04	0.07	0.04
Hearts & Flowers	Flowers accuracy	−0.04	0.07	−0.01
	Flowers RT correct	−0.02	−0.01	0.04
	Flowers RT incorrect	0.18^*^	−0.10	−0.18^*^
	Flowers Δ RT	0.07	−0.05	−0.01

Spearman correlations. Significant correlations are denoted with asterisk: ^*^p < 0.05, ^***^p < 0.001.

We found limited correlations between behavior types and measures of inhibition at pre-test. On-task behavior correlated with RT on incorrect trials (ρ = 0.18, *p* = 0.042), thus on-task behavior was more frequent in children with longer RT on incorrect trials; that is, those who commited fewer impulsive errors. The opposite relationship was found for active off-task behavior, which was more frequent in children with shorter RT on incorrect trials (i.e., those who made more impulsive errors; ρ = −0.18, *p* = 0.037). Table 4 includes all correlations between behavior and measures of inhibition.

### 3.2 Physical activity and inhibition

**Figure 5:**
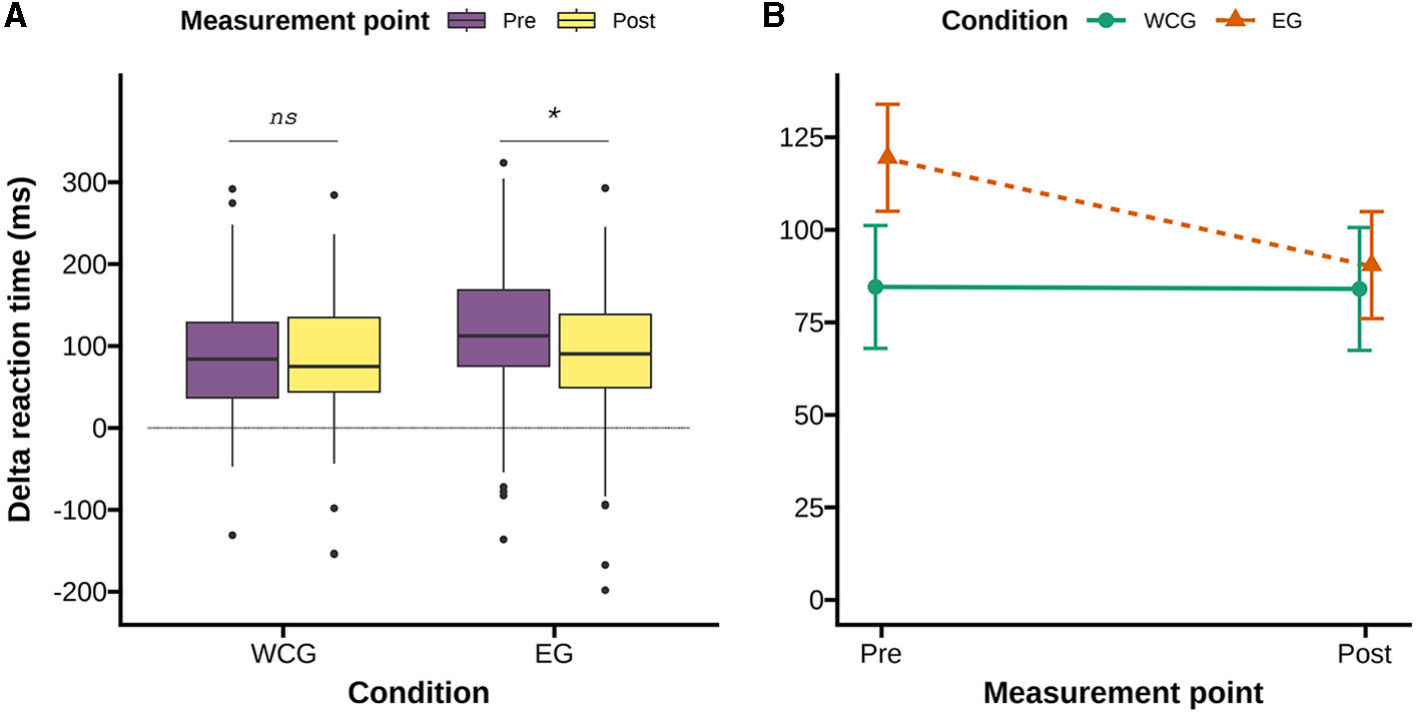
Inhibition measures at pre- and post-test, by condition. **(A)** Pairwise comparisons. **(B)** Group difference in improvement from pre- to post-test. WCG, waitlist control group; EG, experimental group. ^*^p < 0.05, ^**^p < 0.01, ^***^p < 0.001.

For Δ RT in the Flowers block, significant main effects of time [*F*_(1, 209)_ = 5.76, *p* = 0.017] and condition [*F*_(1, 209)_ = 5.68, *p* = 0.018] were found, along with a time-by-condition interaction [*F*_(1, 209)_ = 4.03, *p* = 0.046]. *Post-hoc* pairwise contrasts indicated that at post-test, the Δ RT was significantly smaller than at pre-test in the EG [*t*_(209)_ = 3.12, *p* = 0.011], but not in the WCG [*t*_(209)_ = 0.05, *p* = 0.999; Figure 5A]. Follow-up analysis of the rate of change from pre- to post-test indicated a significant difference between the EC and WCG [*t*_(209)_ = 2.01, *p* = 0.046; Figure 5B]. The effect size for the interaction effect was small (ω^2^ = 0.01).

**Figure 6:**
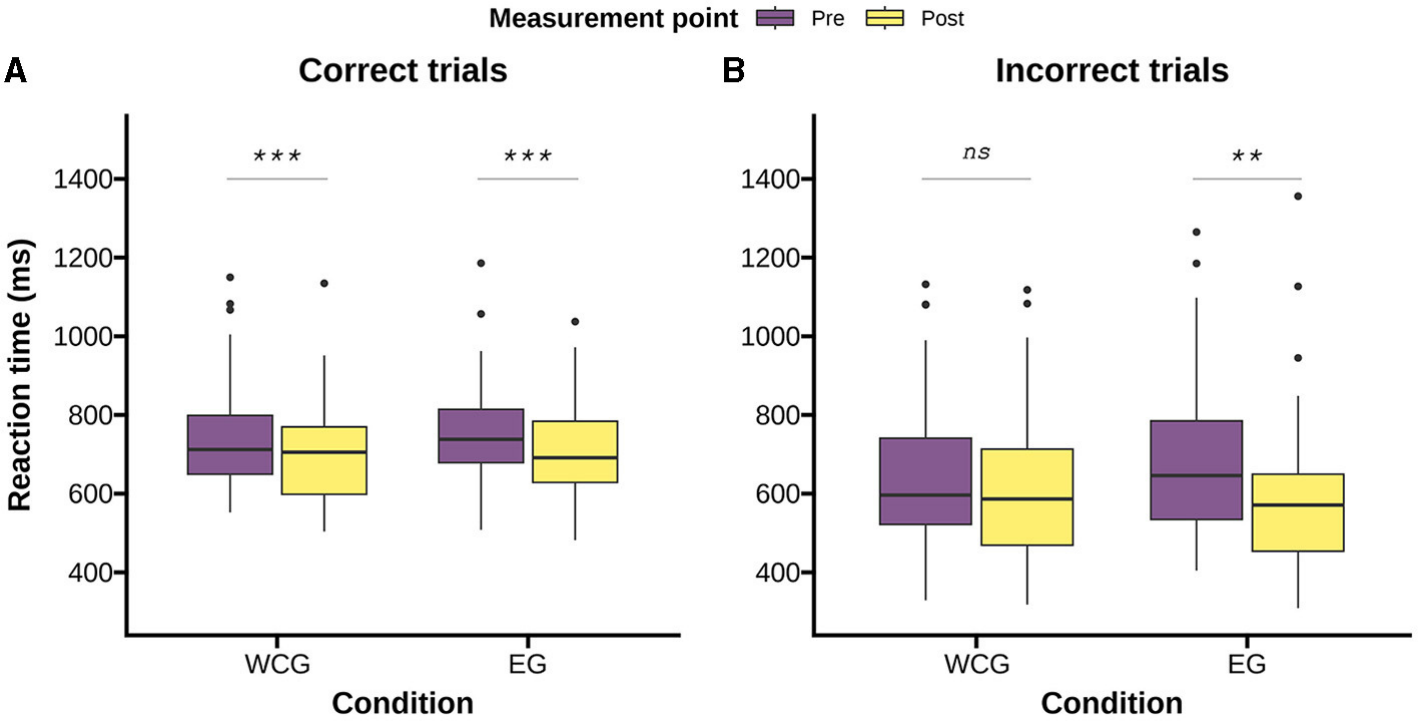
Reaction time change in HF task, from pre- to post-test, by condition. **(A)** Correct trials, **(B)** Incorrect trials. WCG, waitlist control group; EG, experimental group. ^*^p < 0.05, ^**^p < 0.01, ^***^p < 0.001.

A significant main effect for time was also found for RT on correct as well as incorrect trials [correct: *F*_(1, 209)_ = 40.26, *p* < 0.001; incorrect: *F*_(1, 84)_ = 9.06, *p* = 0.004], with RT at post-test being shorter than at pre-test. The extent of the change in RT did not differ between the conditions, as we found no interaction effect for time and condition [correct trials: *F*_(1, 209)_ = 0.43, *p* = .511, Figure 6A; incorrect trials: *F*_(1, 84)_ = 0.62, *p* = 0.434, Figure 6B].

### 3.3 Physical activity and task-related classroom behavior

**Figure 7:**
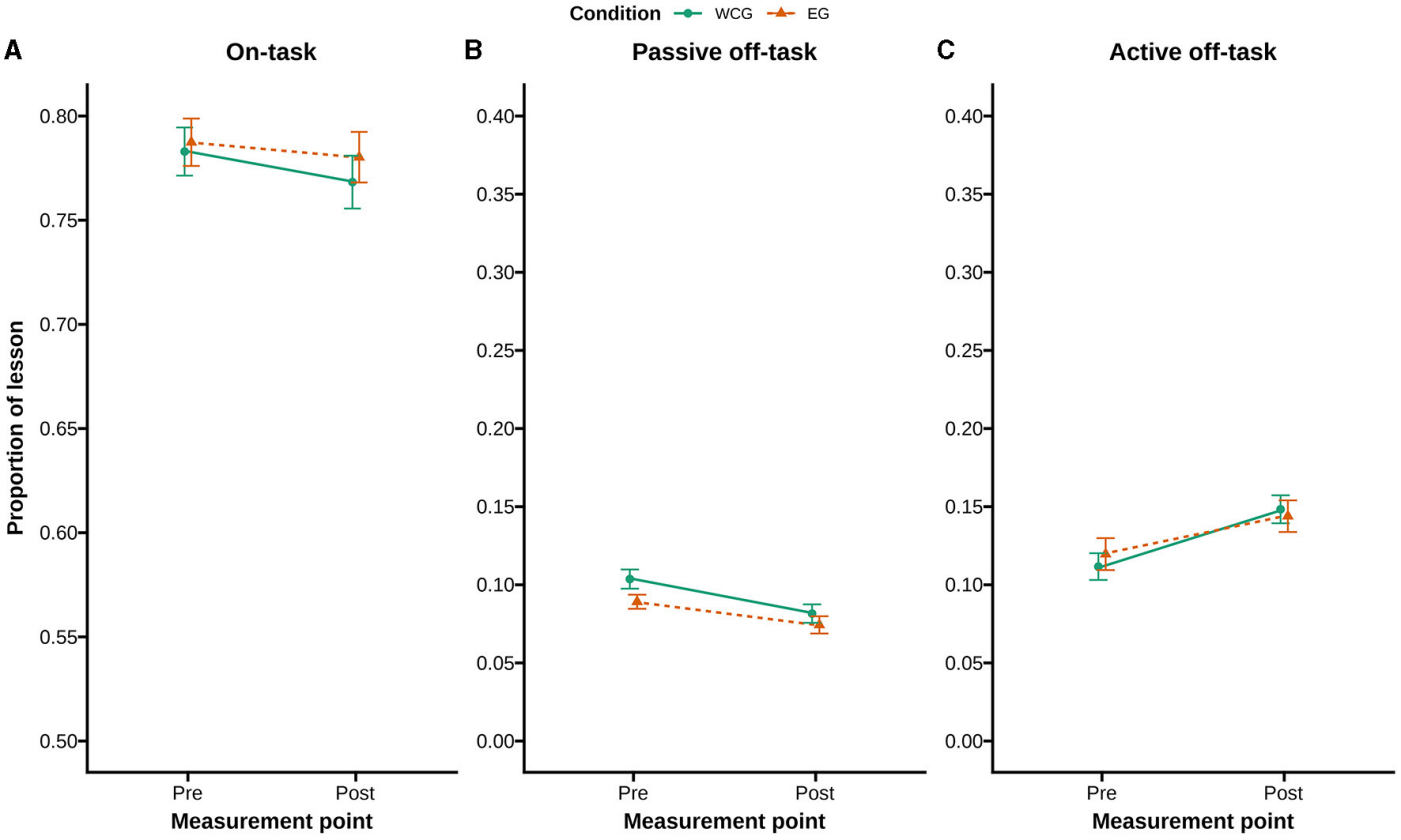
Task-related classroom behavior proportions at pre- and post-test, by condition. **(A)** On-task behavior. **(B)** Passive off-task behavior. **(C)** Active off-task behavior.

Multilevel models indicated that on-task and passive off-task behavior did not change meaningfully from pre- to post-test in either group. The probability of on-task behavior occurring declined in both groups (see Figure 7A). The decrease was less pronounced in the EG (0.3% points vs. 2.4% points in the WCG, see Table 5), yet both declines were negligible (EG: *d* = −0.01, WCG: *d* = −0.15). At the participant level, the intercept correlated positively with the slope of the interaction term (0.30), indicating that those children who were more on-task at pre-test experienced a stronger effect of the intervention, thus more strongly preventing a decline in their on-task behavior.

**Table 5:** Model results logistic three-level regressions.

**Condition**	**Measurement**	**Predicted**	**95 % CI**	**Odds ratio**	***d***
WCG	Pre	On-task behavior			
		0.916	0.857–0.952	0.76	−0.15
	Post	0.892	0.821–0.937		
		
EG	Pre	0.902	0.839–0.942	0.98	−0.01
	Post	0.899	0.835–0.940		
		
WCG	Pre	Passive off-task behavior			
		0.053	0.030–0.092	0.90	−0.06
	Post	0.048	0.026–0.084		
		
EG	Pre	0.052	0.031–0.088	0.74	−0.17
	Post	0.039	0.022–0.069		
		
		Active off-task behavior			
WCG	Pre	0.022	0.009–0.054	1.68	0.29
	Post	0.036	0.015–0.085		
		
EG	Pre	.027	0.011–0.062		
	Post	0.034	0.015–0.080		
				1.31	0.15

WCG, waitlist control group; EG, experimental group.

The probability of passive off-task behavior marginally decreased in both groups (see Figure 7B); in the WCG, the probability of passive off-task behavior was half a percentage point lower at post-test, whereas for the EG, it was 1.3% points lower. Again, both decreases failed to reach practical significance (*d* = −0.06 and *d* = −0.17 respectively, see Table 5). The participant level intercept-slope interaction for passive off-task behavior was positive (0.28), now indicating that those children who displayed more passive off-task behavior at pre-test experienced a greater effect of the intervention, whereby they more strongly reduced their passive off-task behavior.

The probability of active off-task behavior occurring significantly increased from pre- to post-test in the WCG (1.4% points, *d* = 0.29, small effect) but not in the EG (0.7% points, *d* = 0.15, negligible; see Table 5 and Figure 7C). The intercept-slope interaction for active off-task behavior at the participant level was also positive (0.27). Thus, children who were the most actively off-task at pre-test experienced a stronger effect of the intervention and more strongly prevented their active off-task behavior increasing at post-test.

### 3.4 Mediation effects

We found no mediation effect for inhibition. The Δ RT was the only measure of inhibition to respond to PA in the ANOVA, and was added to the MLM as the mediator. For on-task classroom behavior, the total effect of PA was *b*_logit_ = 0.25, and the indirect effect of PA via inhibition was *b*_logit_ = −0.01, indicating a non-significant mediation effect of PA on on-task behavior via inhibition (*P*_*M*_ = −0.05). For the off-task classroom behaviors, similar patterns of effects were found, with mediation effects failing to reach practical significance (passive off-task: *P*_*M*_ = −0.14, active off-task: *P*_*M*_ = −0.03; see Table 6). As inhibition improved after PA, but classroom behavior still worsened over time (although less strongly so in the EG than in the WCG), the direction of the indirect effect is opposite to the total effect, and thus the value of *P*_*M*_ is negative.

**Table 6:** Mediation effect of inhibition.

**Classroom behavior**	**Total effect of PA**	**Direct effect of PA**	**Indirect effect of PA**	**Mediation effect**
	*b*_logit_	*b*_logit_	*b*_logit_	*P*_*M*_
On-task	0.25	0.26	−0.01	−0.05
Passive off-task	−0.18	−0.21	0.03	−0.14
Active off-task	−0.25	−0.26	0.01	−0.03

## 4 Discussion

This study aimed to investigate the effects of a short, intermittent bout of PA aimed at high-intensity with high complexity on inhibition and task-related classroom behavior in fourth grade children. Moreover, we examined the relationship between inhibition and task-related classroom behavior and explored a possible mediation of the effects of PA on task-related classroom behavior by inhibition. We expected to find a positive effect of PA on inhibition and task-related classroom behavior, as well as a small correlation between inhibition and task-related classroom behavior.

### 4.1 Physical activity and inhibition

We found that inhibition improved in the EG, but not the WCG, with a small effect size (ω^2^ = 0.01), thus confirming our first hypothesis. This is in line with findings reported in the literature (e.g., Ma et al., 2015; Latomme et al., 2022), although the size of the effect in the present study is somewhat smaller than those reported by Jäger et al. (2014), who found a moderate effect of a 20-min cognitively engaging PA break on inhibition (η_p_^2^ = 0.06) in their sample of 6- to 8-year-old children and Martins et al. (2021), who found large effects (η_p_^2^ = 0.15) of a bout of intermittent high intensity cycling on inhibition in preadolescent children (mean age 10.3 years).

The main difference between the present study and those by Jäger et al. (2014) and Martins et al. (2021) is the group size during intervention administration. Jäger et al. (2014) conducted the exercise sessions in groups of four children, with the cognitive tests carried out in individual settings. Martins et al. (2021) do not specify the group size for administration, yet randomization was carried out at the individual level and participants' HR was monitored on a one-to-one basis by research staff. These more closely controlled conditions for PA and the cognitive test execution may have helped to increase the benefits of the PA for cognitive skills, yet it does not correspond to an ecologically valid classroom situation. Thus, the findings in the present study may have been affected by contextual influences. However, still detecting a small effect size in an ecologically valid classroom scenario is a significant result. This suggest that, when PA is administered by a single member of staff to an entire class, the benefits to cognition are still noticeable.

### 4.2 Physical activity and task-related classroom behavior

We could confirm our hypothesis that PA improves task-related classroom behavior, but only for active off-task behavior. As active off-task behavior tends to also disrupt others, this is an important aspect of classroom behavior. Unfortunately, we did not find significant effects of PA on passive off-task or on-task behavior.

#### 4.2.1 Off-task classroom behavior

Only a few studies have reported off-task classroom behavior in relation to PA. Our findings are in line with a study by Heemskerk et al. (2022b), reporting that, after high-intensity PA, active off-task behavior was significantly reduced with a similar effect size to the present study (small effects in both studies) and passive off-task behavior was not significantly affected. In another study reporting on off-task behavior, Ma et al. (2014) reported small effects of PA on passive (ES = 0.31) and motor (ES = 0.48) off-task behaviors, but not on verbal off-task behavior. In the present study, motor and verbal off-task behavior were both included in the “active off-task” category, for which a small deterioration (ES = 0.29) was identified in the WCG, but not in the EG. The inclusion of both verbal and motor off-task behaviors may have reduced the effect size in the present study (0.29 vs. 0.48 for Ma et al., 2014).

The finding that PA prevented an increase in active off-task behavior in the present sample rather than reducing it may be related to the very low occurrence of active off-task behavior at pre-test (11% for WCG and 12% for EG), possibly indicating a floor-effect. The occurrence of passive off-task was of similar magnitude at 11 and 9%, respectively. Thus, a reduction was possible, yet with variance at post-test of σ^2^ = 0.005 and σ^2^ = 0.007 for the WCG and EG, respectively, no statistical or practical significance was reached. In contrast, Ma et al. (2014) report far higher occurrences of off-task classroom behavior in their baseline condition at 37% for active off-task behavior and 23% for passive off-task behavior, and Mavilidi et al. (2020) report baseline off-task behavior between 20% and 34%. In sum, although low levels of off-task behavior are positive, they might have limited the detection of an intervention effect in the present intervention. And, the practical implication of our findings is that when a teacher notices an increase in off-task behavior in their classroom, they can remedy this by introducing a short bout of cognitively demanding PA aimed at high-intensity.

#### 4.2.2 On-task classroom behavior

We found that on-task classroom behavior decreased at post-test in both groups, with the decrease in the EG somewhat less pronounced than in the WCG. Our results are similar to those reported by Goh et al. (2016), in that on-task behavior decreased at post-test in the control group. However, in the present sample, on-task behavior in the EG still slightly reduced from pre- to post-test, whereas in the study by Goh et al. (2016) an increase in on-task behavior was observed after PA. Similar to the floor effect for active off-task behavior, there may have been ceiling effects in the on-task behavior of the present sample. At 79%, on-task behavior at pre-test in the present sample was notably higher than in other reports of the effects of PA on on-task behavior.

For example, Mahar et al. (2006) report an effect size of ES = 0.6 on a baseline of around 70% on-task behavior. Moreover, for those with lower levels of on-task behavior at pre-test the effect size was much larger at ES = 2.2. Likewise, Heemskerk et al. (2020) reported on-task behavior to be around 74%, and identified an increase in on-task behavior with a small effect size for VPA with high complexity (ES = 0.33), and Heemskerk et al. (2022b) noted that for the 33% of the sample with the lowest on-task behavior the effect of PA at any intensity was positive (ES > 0.40) with a large effect size for highest intensity PA sessions (ES = 0.71). Finally, Mavilidi et al. (2020) report adjusted mean differences between 27.0% (MPA vs. control) and 44.1% (MPA with maths input vs. control). However, they differentiated between active and passive on-task behaviors, resulting in much lower baseline scores (between 26 and 52%) and far greater scope for improvement (Mavilidi et al., 2020).

Thus, considering the very high level of on-task classroom behavior in the current sample, it is not surprising that rather than an improvement, a stabilization and prevention of decline were identified. This also aligns with the intercept-slope interaction found, indicating that those with the highest level of on-task behavior at pre-test experienced a greater prevention of the decline at post-test. In practical terms, when teachers detect a decline in levels of on-task behavior in their classroom, a short cognitively demanding PA break aimed at high-intensity can be used to bring their students' focus back to the lesson.

### 4.3 Mediation of task-related behavior by inhibition

We found no evidence of mediation of the effect of PA on task-related behavior by inhibition in the present sample (*P*_*M*_ = −0.03 to −0.14).

This may be because PA does not affect behavior and inhibition equally, as these constructs are related, yet separate. In younger children (pre-K to 2nd grade), the relationship between EF and academic achievement was found to be mediated by on-task behavior (Nesbitt et al., 2015; McCoy et al., 2022; Heemskerk and Roebers, 2023), thus indicating that EF does play a role in predicting on-task behavior. This relationship between EF and behavior suggests that the two constructs have shared variance, but also that they have unique aspects, which do not overlap. Indeed, in a study by Mavilidi et al. (2020), effects of PA breaks on behavior were found, but not on EF (inhibition and WM). This is in line with conclusions of a meta-analysis by Masini et al. (2020) that behavioral effects of PA are more consistently identified than EF effects.

It thus is possible that, despite an overlap in the mechanisms by which PA influences EF and task-related behavior, these influences differ in strength, vary in their responsiveness to various parameters of PA, and occur alongside other processes in a classroom environment. For example, Heemskerk et al. (2022a) found that the amount of MVPA influenced how tired children felt after their PA session, but tiredness did not influence their task-related classroom behavior. Yet, negative effects of tiredness on cognitive functions have been identified in several studies (e.g. Hockey, 1997; Ishii et al., 2014). Types of PA which induce tiredness may therefore have differential effects on EF and task-related behavior.

It may also be that the mediating or predictive role of inhibition for task-related behavior is sensitive to the participants' level of inhibition. Previous studies reporting relationships between inhibition and task-related behavior have had younger samples (pre-K to 2nd grade) (Nesbitt et al., 2015; McCoy et al., 2022; Heemskerk and Roebers, 2023). In the present sample, inhibition accuracy was particularly high at 96%. It may be that the effects of inhibition on task-related behavior were limited by inhibition being generally well-developed, and other factors were therefore more predictive of variations in task-related behavior. The measure of inhibition derived from the HF task is specific to the inhibition of in-task distractors and pre-programmed responses. Yet, in a classroom, distractions are often environmental. Moreover, the involvement of peers may make these environmental distractions more (emotionally) valiant and add social aspects to the ability to ignore these task-irrelevant stimuli and maintain task focus.

We did find a correlation between impulsive responses and active off-task behavior—the type of behavior which is most disruptive in classrooms. This is in line with research conducted by Berlin et al. (2004) in a sample of children with ADHD. Berlin et al. (2004) found that poor inhibition—measured as errors of commission on the go/no-go task—correlated with both inattention and disruptive behavior at school. We did not, however, find an affect of the PA break on this measure of inhibition. It may be that in our study, we did not optimize the PA parameters for both inhibition *and* task-related classroom behavior.

Most research into the effects of PA on inhibition uses the RT for correct trials or the Δ RT score as their outcome measure, which we found to respond differentially to our PA break. This is in line with recent studies finding that various measures of EF optimally respond to different types of PA content. Firstly, Wang et al. (2023) found that, for example, RT for inhibition tasks was improved most strongly by ball games, whereas inhibition accuracy responded most favorably to cognitively engaging PA (Wang et al., 2023). Similarly, Kao et al. (2023) report processing speed (measured by RT) to be improved by HIIT training, whereas attention benefited most from moderate intensity PA.

Promising results have been found for studies using adaptive protocols, such as the exergaming study by Anzeneder et al. (2023). In their intervention, the physical intensity of the activity was continuously adapted on the basis of the participants' heart rate, whilst the cognitive complexity was adapted as a function of errors made. However, although such adaptive activities allow for more precise investigations of the optimum level of intensity and complexity of PA for cognitive benefits, they require costly equipment and are not feasible for sustained whole-class activity in the majority of school settings. For future research into tailoring even better PA break however, such adaptive procedures might be very promising.

A final possible avenue is that, rather than a mediation effect, there is a more complex interrelation of parameters at play. For example, we found that while some measures of inhibition responded to PA (slowing down of RT, reflected by Δ RT), others correlated with task-related behavior (impulsive errors, reflected by RT on incorrect trials). Additionally, a moderation effect of baseline inhibition or the magnitude of the improvement of inhibition may play a role. In a mediation model, such as the one used in the present study, those participants who did not improve their inhibition score may suppress the detection of the role of inhibition in the relationship between PA and task-related behavior. Moderation of the effects of PA have previously been identified in several studies where the effects of PA on cognition at the sample level were masked, including by the amount of MVPA accrued (e.g., Gilbert et al., 2023), and the level of academic achievement or cardio-respiratory fitness of the participants (e.g., Jäger et al., 2015). Thus, further investigations of the interrelations of PA, inhibition, and task-related in primary school children, and differential effects of interventions are called for.

### 4.4 Strengths and limitations

The major strength of this study—its ecological setting in the classroom—brings some limitations in terms of the measurement of PA intensity. The PA break was designed to be quick and simple, with minimal disruption to the daily schedule, so that it should be feasible for classroom teachers to carry out. In an everyday situation in school, teachers would encourage all students in the class to take part fully, but would not be able to monitor device-based intensity level for an entire class. Thus, we did not use devices and reduced the burden of setting up the session or clearing up afterwards. This increase in ecological validity means that we are unable to use PA intensity as a variable in our statistical models. A second limitation for the statistical modeling was the high level of on-task behavior as well as performance on the inhibition task at pre-test. This may have limited our ability to establish effects of the PA break on these outcomes. Future studies in this age group should ensure that they employ a sufficiently difficult task to measure inhibition and avoid ceiling effects. A further strength of this study is the concurrent collection of behavior and inhibition data in relation to PA in primary school children. Although both constructs have been linked to PA, and the underlying mechanisms responsible for these effects are thought to overlap, unfortunately, only a handful of studies have yet been published which concurrently investigate this.

### 4.5 Implications

Our results show that, for teachers, a short PA break aimed at high intensity PA with cognitive demands can improve inhibition and task-related classroom behavior in an easy and time-efficient manner. Additionally, this study contributes to the literature aiming to uncover the mechanisms by which PA affects different aspects of cognition, including a decrease in off-task classroom behavior, as well as the relationships between these aspects of cognition. It appears that, although inhibition and task-related classroom behavior share some variance and are both improved by PA, the effect of PA on task-related behavior is not mediated by inhibition.

## Data availability statement

The datasets presented in this study can be found in online repositories. The names of the repository/repositories and accession number(s) can be found at: https://doi.org/10.17605/OSF.IO/ETHXS.

## Ethics statement

The studies involving humans were approved by University of Bern, Institute of Psychology. The studies were conducted in accordance with the local legislation and institutional requirements. Written informed consent for participation in this study was provided by the participants' legal guardians/next of kin.

## Author contributions

CH: Conceptualization, Data curation, Formal analysis, Investigation, Methodology, Project administration, Visualization, Writing – original draft, Writing – review & editing. CR: Conceptualization, Resources, Supervision, Writing – original draft, Writing – review & editing.
